# Early malaria infection, dysregulation of angiogenesis, metabolism and inflammation across pregnancy, and risk of preterm birth in Malawi: A cohort study

**DOI:** 10.1371/journal.pmed.1002914

**Published:** 2019-10-01

**Authors:** Robyn E. Elphinstone, Andrea M. Weckman, Chloe R. McDonald, Vanessa Tran, Kathleen Zhong, Mwayiwawo Madanitsa, Linda Kalilani-Phiri, Carole Khairallah, Steve M. Taylor, Steven R. Meshnick, Victor Mwapasa, Feiko O. ter Kuile, Andrea L. Conroy, Kevin C. Kain

**Affiliations:** 1 Sandra Rotman Centre for Global Health, University Health Network-University of Toronto, Toronto, Ontario, Canada; 2 College of Medicine, University of Malawi, Blantyre, Malawi; 3 Department of Clinical Sciences, Liverpool School of Tropical Medicine, Liverpool, United Kingdom; 4 Department of Epidemiology, Gillings School of Global Public Health, University of North Carolina at Chapel Hill, Chapel Hill, North Carolina, United States of America; 5 Division of Infectious Diseases and Duke Global Health Institute, Duke University, Durham, North Carolina, United States of America; 6 Department of Pediatrics, Indiana University School of Medicine, Indianapolis, United States of America; Mahidol-Oxford Tropical Medicine Research Unit, THAILAND

## Abstract

**Background:**

Malaria in pregnancy is associated with adverse birth outcomes. However, the underlying mechanisms remain poorly understood. Tight regulation of angiogenic, metabolic, and inflammatory pathways are essential for healthy pregnancies. We hypothesized that malaria disrupts these pathways leading to preterm birth (PTB).

**Methods and findings:**

We conducted a secondary analysis of a randomized trial of malaria prevention in pregnancy conducted in Malawi from July 21, 2011, to March 18, 2013. We longitudinally assessed circulating mediators of angiogenic, metabolic, and inflammatory pathways during pregnancy in a cohort of HIV-negative women (*n* = 1,628), with a median age of 21 years [18, 25], and 562 (35%) were primigravid. Pregnancies were ultrasound dated, and samples were analyzed at 13 to 23 weeks (Visit 1), 28 to 33 weeks (Visit 2), and/or 34 to 36 weeks (Visit 3). Malaria prevalence was high; 70% (*n* = 1,138) had PCR-positive *Plasmodium falciparum* infection at least once over the course of pregnancy and/or positive placental histology. The risk of delivering preterm in the entire cohort was 20% (*n* = 304/1506). Women with malaria before 24 weeks gestation had a higher risk of PTB (24% versus 18%, *p* = 0.005; adjusted relative risk [aRR] 1.30, 95% confidence interval [CI] 1.04–1.63, *p* = 0.021); and those who were malaria positive only before week 24 had an even greater risk of PTB (28% versus 17%, *p* = 0.02; with an aRR of 1.67, 95% CI 1.20–2.30, *p* = 0.002). Using linear mixed-effects modeling, malaria before 24 weeks gestation was associated with altered kinetics of inflammatory (C-Reactive Protein [CRP], Chitinase 3-like protein-1 [CHI3L1], Interleukin 18 Binding Protein [IL-18BP], soluble Tumor Necrosis Factor receptor II [sTNFRII], soluble Intercellular Adhesion Molecule-1 [sICAM-1]), angiogenic (soluble Endoglin [sEng]), and metabolic mediators (Leptin, Angiopoietin-like 3 [Angptl3]) over the course of pregnancy (χ^2^ > 13.0, *p* ≤ 0.001 for each). Limitations include being underpowered to assess the impact on nonviable births, being unable to assess women who had not received any antimalarials, and, because of the exposure to antimalarials in the second trimester, there were limited numbers of malaria infections late in pregnancy.

**Conclusions:**

Current interventions for the prevention of malaria in pregnancy are initiated at the first antenatal visit, usually in the second trimester. In this study, we found that many women are already malaria-infected by their first visit. Malaria infection before 24 weeks gestation was associated with dysregulation of essential regulators of angiogenesis, metabolism, and inflammation and an increased risk of PTB. Preventing malaria earlier in pregnancy may reduce placental dysfunction and thereby improve birth outcomes in malaria-endemic settings.

## Abstract 2

In this cohort study, Robyn Elphinstone and colleagues investigate if malaria infection in early pregnancy may be associated with changes in placental growth and function, as well as preterm birth.

## Author summary

**Why was this study done?:**

- Malaria infection during pregnancy has serious consequences for the mother and her unborn child.
- We have a poor understanding of the mechanisms by which malaria causes adverse birth outcomes, especially preterm birth (PTB), a leading cause of death in children less than 5 years of age.
- Efforts to date to prevent malaria in pregnant women have not been shown to reduce the risk of PTB associated with malaria infection.
- We conducted this study to assess if malaria infection, especially early in pregnancy, might alter important factors that control placental growth and function and determine if these lead to PTB.

**What did the researchers do and find?:**

- We measured levels of 10 different angiogenic, inflammatory, and/or metabolic proteins longitudinally during pregnancy in a large cohort of 1,628 Malawian women at risk of malaria infection and demonstrate that early infection with malaria is associated with changes in several key proteins that are important for healthy pregnancies.
- The use of ultrasound dating highlighted the contribution of early malaria infection to an increased risk of delivering preterm, previously thought to occur primarily from malaria late in pregnancy.

**What do these findings mean?:**

- Current interventions to prevent malaria in pregnancy are usually initiated at the first antenatal visit in the second trimester.
- This study demonstrates that many women are already malaria-infected at this first visit and that these early infections are sufficient to alter factors essential for the placenta to adequately support fetal growth and healthy pregnancy outcomes.
- These early infections increase the risk of delivering preterm, indicating that interventions to prevent malaria in pregnancy may need to be started earlier in pregnancy in order to reduce malaria-associated adverse birth outcomes.

## Introduction

In sub-Saharan Africa, an estimated 28 million women were at risk of malaria in pregnancy (MIP) in 2014 [1]. MIP is associated with adverse birth outcomes, including preterm birth (PTB), fetal growth restriction (FGR), and stillbirth [2–4]. However, our understanding of the underlying mechanisms is limited [2]. Low birth weight (LBW) infants, including those caused by PTB and those born small for gestational age (SGA), are at increased risk of death during infancy with the risk extending to all-cause mortality in adulthood [5–7]. Furthermore, LBW infants are at increased risk of developing chronic illnesses, including diabetes, hypertension, cancer, and neuropsychiatric disorders [5,8,9].

The timing of MIP may influence the risk and type of adverse birth outcome. The risk of FGR appears to be higher in women with antenatal malaria earlier in pregnancy [10–14]. In contrast, women with PTB are reported to be at higher risk if they had malaria later in pregnancy [13,14], including at delivery [10,11]. However, mechanistically, it is unclear how the timing of MIP differentially impacts birth outcomes.

Following red-cell invasion, the parasite expresses *Plasmodium falciparum* erythrocyte membrane protein 1 (PfEMP1) on the surface of the infected erythrocyte. During pregnancy, a PfEMP1 variant encoded by *var2csa* mediates adhesion of the infected erythrocyte to chondroitin sulphate A and enables it to sequester within the placental intervillous space, contributing to inflammatory infiltrates and reduced nutrient transfer to the fetus [2,15]. Using a combination of structure equation modeling and in vivo models of MIP, Conroy and colleagues showed that monocytic infiltration contributes to complement activation and subsequent dysregulation of angiogenic factors, such as soluble Endoglin (sEng), that are essential for placental angiogenesis and vascular remodeling [16]. Although a detailed understanding of the mechanisms underlying malaria-associated adverse birth outcomes is lacking, the available evidence indicates that malaria infection may alter placental vascular development as evidenced by increased placental vascular resistance and micro-CT imaging of placental vasculature in human studies and preclinical models, respectively [16–24]. Previous pregnancy studies have shown that alterations in angiogenic and inflammatory mediators are associated with adverse birth outcomes at delivery [18,19,25]. However, these observations need to be evaluated over the course of pregnancy in a large cohort of women with high levels of malaria exposure.

We hypothesize that malaria early in pregnancy results in the dysregulation of inflammatory and angiogenic pathways resulting in placental insufficiency and adverse birth outcomes. To test this hypothesis, our objective was to longitudinally characterize circulating levels of angiogenic, metabolic, and inflammatory mediators over the course of pregnancy in a large cohort of Malawian women in an area with moderate to intense malaria transmission and assess the association of malaria infection with adverse birth outcomes.

## Methods

### Study population and trial design

This study cohort was nested within a randomized clinical trial of MIP prevention in Malawi. Briefly, HIV-negative women enrolled from July 21, 2011, to March 18, 2013 were randomized to intermittent preventive treatment in pregnancy (IPTp) with sulfadoxine-pyrimethamine (SP), the standard of care, or intermittent screening and treatment in pregnancy (ISTp) with dihydroartemisinin-piperaquine (DP) [26].

Participants were eligible for inclusion in the trial if they were HIV negative, singleton pregnancies, agreed to deliver in a local health facility, hemoglobin >70 g/L, and had not previously received IPTp-SP [26]. Participants were eligible for the present study if they were enrolled <24 weeks of gestation, and had frozen plasma samples for testing from one or more of the following gestational ages: 13 to 23 weeks (Visit 1), 28 to 33 weeks (Visit 2), 34 to 36 weeks (Visit 3). The study size was determined based on availability of samples from the parent trial. Participants were excluded if no plasma samples were available for testing or if there were no PCR or placental histology data available. All women were provided with an insecticide-treated bed net at their enrolment visit.

The ultrasound dating was performed during the second trimester on a Sonosite S180 portable ultrasound scans (Sonosite, Bothell, Washington) by M. Madanitsa and research nurses as described by Wylie and colleagues [27]. The estimated gestational age was calculated by the Hadlock formula. The majority of deliveries were either spontaneous or assisted (by vacuum or forceps); however, there were a small number of deliveries performed by C-section. No data were available on inductions or indications for C-sections in this cohort.

This study is reported as per the Strengthening the Reporting of Observational Studies in Epidemiology (STROBE) guideline (S1 STROBE Checklist). All methods of assessment were similar across groups, and malaria status and birth outcomes were unknown to the individuals performing the mediator assays.

### Plasma marker testing

Angiogenic, inflammatory, and metabolic factors tested were selected based on previous studies [18,19,25]. Luminex multiplex assays or ELISAs were used to measure plasma factor concentrations (R&D Systems. Minneapolis, MN; S1 Table). The following mediators were tested by Luminex assays: Leptin, sEng, Placental Growth Factor (PlGF), and soluble Fms-like Tyrosine Kinase-1 (sFlt-1/soluble VEGFR1; Custom kit); soluble Intercellular Adhesion Molecule-1 (sICAM-1), Angiopoietin-like 3 (Angptl3), Chitinase 3-like protein-1 (CHI3L1), and soluble Tumor Necrosis Factor receptor II (sTNFRII; Kit # LXSAHM-5). Data were collected using xPONENT version 4.2 software on a Luminex MagPix machine (Luminex, Toronto, Canada). ELISAs were used to measure C-Reactive Protein (CRP) and Interleukin 18 Binding Protein (IL-18BP; DuoSet ELISA Kits), modified protocol as previously reported by Conroy and colleagues [28].

### Definitions

Adverse birth outcomes were defined as LBW (birth weight <2,500g), PTB (<37 weeks gestational age based on ultrasound dating, completed during the second trimester), SGA (<10th percentile for gestational age using Intergrowth 21st Standards [29]), and nonviable births (either stillbirth [>28 weeks] or spontaneous abortion [<28 weeks]). PTB, LBW, and SGA were dichotomous variables based on standard clinical case definitions as above [4]. Educational status was defined by years of schooling: low (<5), medium (5 to <10), or high (≥10). Socioeconomic status was defined in tertiles using principal component analysis based on a survey including various household assets and characteristics. Socioeconomic status and educational status were kept continuous when adjusted for in multivariate and/or multivariable models. Data on rates of preeclampsia were unavailable for this cohort. Malaria status was assessed using real-time PCR during pregnancy, and histology and/or PCR at delivery; the real-time PCR assay used targeted both the parasite gene *pfldh* and the human gene beta-tubulin as an internal control. PCR detection was completed for specimens from scheduled visits (up to 5 throughout pregnancy: 13–23 weeks, 24–27 weeks, 28–33 weeks, 34–36 weeks, and at delivery) and from any unscheduled sick visits, up to 8 visits for some women [26]. Malaria-negative women were defined as women without any recorded positive PCR samples during pregnancy and were negative for malaria by placental histology. Women were defined as “any malaria positive” if they had any malaria-positive PCRs and/or malaria-positive placental histology.

### Ethics

This study was approved by the Liverpool School of Tropical Medicine, the Malawian National Health Science Research Committee, and the University Health Network Research Ethics Committee of the University of Toronto. Written informed consent was obtained for all study participants. The larger trial was registered: Pan African Clinical Trials Registry PACTR201103000280319; ISRCTN Registry ISRCTN69800930.

### Statistical analysis

Statistical analysis was conducted using GraphPad Prism 7, IBM SPSS Statistics 24, and R version 3.5.1 (R Core Team, 2018). A prospective protocol for analysis was not prepared for this study; however, the statistical approach described below was decided prior to commencement of analysis and was based on publications analyzing similar longitudinal data [22,30]. Comparison between continuous variables was done by Mann-Whitney analysis, and comparison between categorical variables was done by Chi-square analysis. For marker comparison based on malaria status at each visit, an adjusted *p* ≤ 0.005 was used to correct for multiple comparisons. Relative risk (RR) and corresponding 95% confidence intervals (CIs) of adverse birth outcomes based on malaria status were calculated using log-binomial regression with a log link function. We used multivariable quantile regression to further assess the effect of malaria infection on gestational age at delivery. Multivariable models were adjusted for treatment arm (ISTp versus IPTp), maternal age, gravidity, socioeconomic status, education status, body mass index (BMI), and hemoglobin at Visit 1. Adjusters were included in multivariable analyses based on an a priori hypothesized relationship with the outcome of interest and were further considered if they had a *p* < 0.05 in bivariate analysis (Table 1). No variables chosen as adjusters by substantive knowledge were excluded based on the results of bivariate analysis. Ultimately, all variables included in the models, including those identified by bivariate screening, have been documented as risk factors for adverse birth outcomes and related to MIP.

**Table 1:** Clinical characteristics of women based on malaria status.

Characteristics of cohort		Malaria negative*		Any malaria positive^@^		
Clinical characteristics		Total (*n*)^&^	*n* (%) or Median [IQR]	Total (*n*)^&^	*n* (%) or Median [IQR]	*P* value^#^^,^ ^$^
Study Arm (IPTp)		490	254 (52)	1,138	557 (49)	0.285
Age (years)		490	23 [20–27]	1,138	20 [18–24]	**<0.001**
Primigravid		490	114 (23)	1,138	448 (39)	**<0.001**
Socioeconomic status (tertiles)	lowest	489	123 (25)	1,136	418 (37)	**<0.001**
	middle		151 (31)		394 (35)	
	highest		215 (44)		324 (29)	
Education status (tertiles)	lowest	489	148 (30)	1,136	339 (30)	**0.015**
	middle		244 (50)		633 (56)	
	highest		97 (20)		164 (14)	
BMI at Visit 1		448	23.1 [21.4–25.1]	1,038	22.7 [21.1–24.4]	**0.018**
Hemoglobin at Visit 1 (g/dL)		448	11.6 [10.7–12.4]	1,038	11.0 [9.9–11.9]	**<0.001**
Gestational age at ultrasound (weeks)		490	20.6 [18.6–22.3]	1,138	20.1 [18.3–22.1]	0.159
**Adverse birth outcomes**						
Gestational age at delivery		446	38.6 [37.6–39.6]	1,090	38.4 [37.1–39.6]	**0.042**
PTB (<37 weeks)		434	74 (17)	1,072	230 (21)	0.054
Extremely early PTB (<28 weeks)			1 (0.2)		1 (0.1)	0.087
Very PTB (28 to <32 weeks)			1 (0.2)		13 (1.2)	
Moderate to late PTB (32 to <37 weeks)			72 (17)		217 (20)	
Birth weight (kg)		409	3.0 [2.7–3.3]	1,042	2.9 [2.6–3.2]	**<0.001**
LBW (<2,500 g)		409	32 (8)	1,042	124 (12)	**0.024**
SGA (<10th percentile)		409	58 (14)	1,042	180 (17)	0.152
Stillbirth or spontaneous abortion		447	12 (2.7)	1,097	19 (1.7)	0.226

*Malaria negative: All PCR assays and placental histology were negative for malaria.

^@^Malaria positive: At least one PCR test or placental histology was positive for malaria.

^#^*n* (%), Chi-square analysis.

^$^Median [IQR], Mann-Whitney Analysis.

Significant *p*-values (*p* < 0.05) are bolded.

^&^Samples (*n*) available for each variable for analysis.

**Abbreviations:** BMI, body mass index; IPTp, intermittent preventive treatment in pregnancy; IQR, interquartile range; LBW, low birth weight; PTB, preterm birth; SGA, small for gestational age

In order to assess the impact of malaria status at Visit 1 on longitudinal changes in plasma concentrations of angiogenic and inflammatory proteins across gestation, multiple linear mixed-effects models were built for each marker using the lme4 package in R [31,32]. This was performed by adapting the approach used by Romero and colleagues [30] as described by Conroy and colleagues [22]. Models were fitted to each angiogenic, inflammatory, or metabolic mediator measured by ELISA/Luminex as a continuous dependent variable. First, a null model was created for each marker, using the following fixed terms: maternal age, gravidity, socioeconomic status, education status, BMI and hemoglobin at Visit 1, and an interaction term between treatment arm and gestational age. Gestational age was shifted to provide a meaningful intercept, so that the minimum gestational age was zero. A restricted cubic spline of gestational age (using the rms package in R [33]) was included as a main effect and in interaction terms to capture variation across time, with 3 knots placed at the 10th, 50th, and 90th quantile of gestational age, as suggested by Harrell [34]. All models also included a by-participant intercept and by-participant slope as random effects. The impact of early malaria on longitudinal changes in angiogenic and/or inflammatory markers was evaluated in a second model by adding malaria status by PCR at Visit 1 as a main effect to the null model. A third model included the interaction between a spline of gestational age and malaria status at Visit 1 to assess the effect of early malaria on mediator concentrations by gestational age. The estimates and standard error are reported for the final model that included malaria status and the interaction term. The likelihood ratio test was used to compare the 3 models and assess the impact of malaria status (by PCR) at Visit 1 on model fit. Acknowledging the biases introduced by the likelihood ratio test, parameters of model fit (i.e., Akaike information criterion [AIC], Bayesian information criterion [BIC]) are also reported for each of the 3 models. Mediator levels were natural log-transformed for the linear mixed-effects modeling, and residual plots did not show apparent deviation from normality or homoscedasticity. To account for design of parent trial and minor longitudinal differences in mediators by treatment arm, treatment group was included as a covariate in all models. Missing data were excluded from the analyses, and the percentage of missing data is reported (S2 Table).

## Results

### Clinical characteristics and malaria status

**Fig 1:**
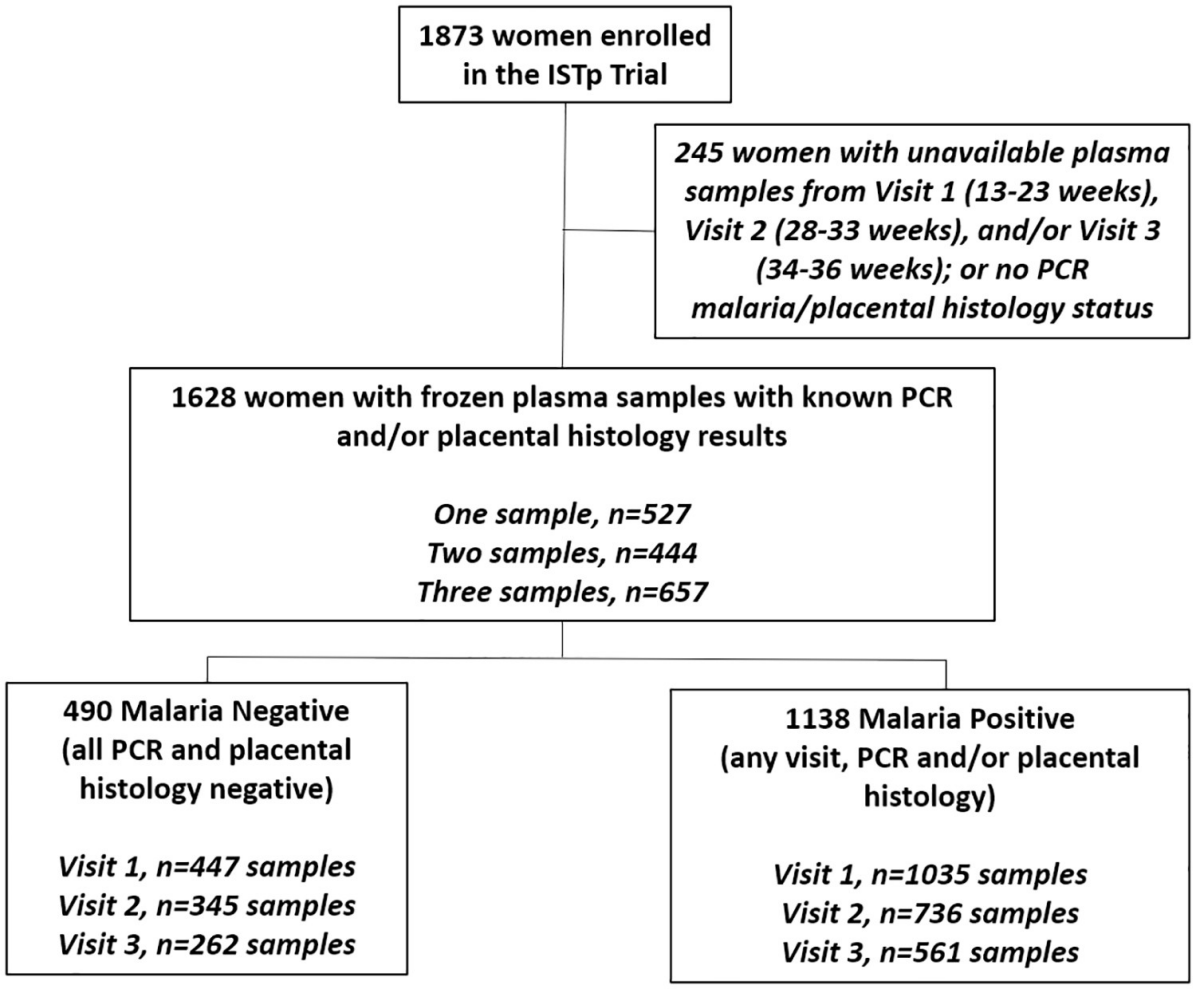
Flow chart for patient population. ISTp, intermittent screening and treatment in pregnancy.

Of the 1,873 women initially enrolled in the trial [26], 3,386 samples were tested from 1,628 women over 3 study visits as shown in Fig 1. The women included in this analysis had a median age of 21 years (interquartile range [IQR] 18, 25), 562 (35%) were primigravid, and 811 (50%) were in the IPTp arm. Of the infants, 11% (*n* = 156/1451) were LBW, 20% (*n* = 304/1506) were preterm, and 16% (*n* = 238/1451) were SGA. Because there were no significant differences between treatment arms in the frequency of malaria at any of the 3 visits, placental malaria, PTB, or SGA, the combined data were analyzed.

A total of 70% (*n* = 1,138) of women had malaria during pregnancy, as determined by at least one positive PCR test for malaria and/or positive placental histology (Table 1). Malaria-positive women were more likely to be younger, primigravid, of lower socioeconomic status, of lower education status, and to deliver at an earlier gestational age with a lower birth weight (Table 1). There were no significant differences in rates of C-section deliveries between malaria-positive women or malaria-negative women (3.0% [*n* = 33] versus 2.5% [*n* = 11], *p* = 0.554). Placental malaria, based on placental histology, was observed in 23% (*n* = 377; Table 2). At Visit 1 (enrolment, prior to treatment allocation; 13 to 23 weeks), 40% (*n* = 649) of women tested positive for malaria (Table 2). Thirty percent (*n* = 490) had no evidence of malaria infection by placental histology and PCR testing at any time during pregnancy.

**Table 2:** Characteristics of malaria infections in the malaria-positive women.

Characteristics	Malaria positive^&^
**Episodes of malaria during pregnancy**	*n* (% of entire cohort)^$^
0*	41 (2.5)
1	464 (29)
2	289 (18)
3	154 (9)
4	97 (6)
5	57 (4)
6	23 (1.4)
7	8 (0.5)
8	4 (0.2)
**Visit 1 (13 to 23 weeks)**^#^	
Malaria positive, peripheral blood	649 (40)
**Visit 2 (28 to 33 weeks)**^#^	
Malaria positive, peripheral blood	192 (12)
**Visit 3 (34 to 36 weeks)**^#^	
Malaria positive, peripheral blood	161 (10)
**Malaria status at delivery**^#^	
Positive malaria, peripheral blood	284 (17)
Positive malaria, placental blood	242 (15)
Placental histology	377 (23)

^&^Malaria positive: At least one PCR test and/or placental histology was positive for malaria.

^$^*n* (%, malaria positive/entire cohort [*n* = 1,628]).

*These women had placental histology positive for malaria.

^#^See S2 Table for numbers of missing data.

### Malaria infection before 24 weeks gestation was associated with an increased risk of preterm birth

**Fig 2:**
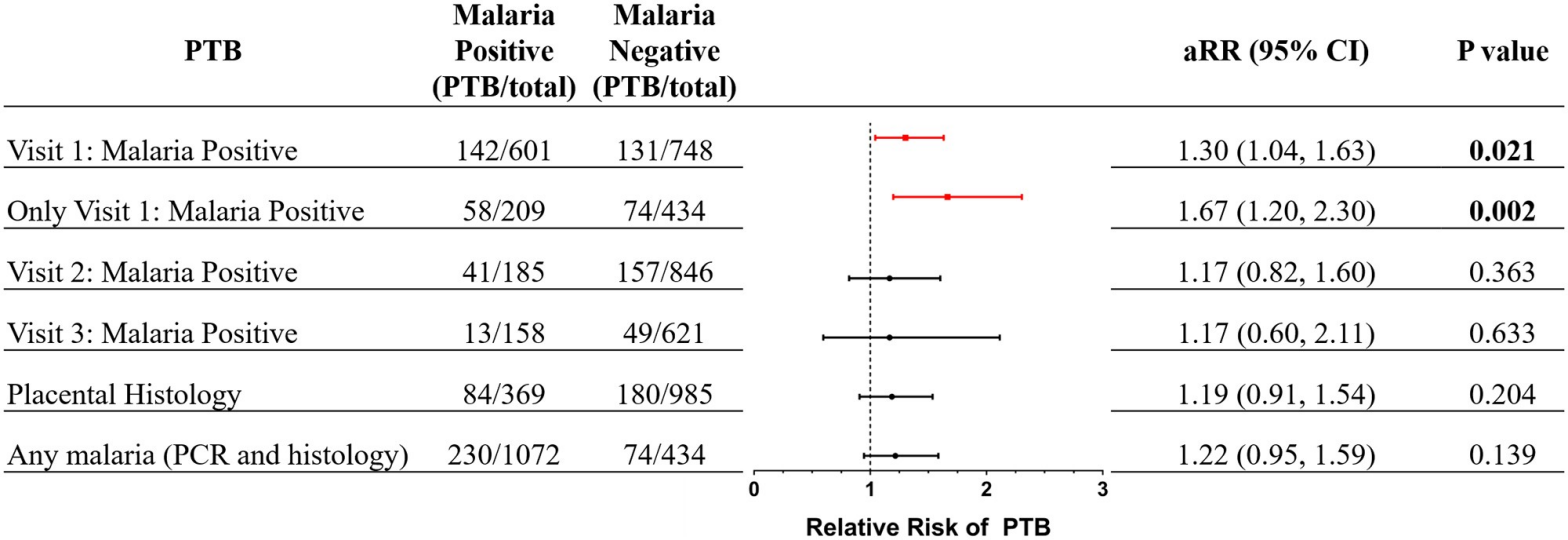
aRR of PTB based on malaria status and/or placental histology. Malaria status of women at each Visit 1 (13–23 weeks), Visit 2 (28–33 weeks) or Visit 3 (34–36 weeks) were assessed. Women who had only a single positive PCR recorded over the course of pregnancy and that positive result was at Visit 1 were denoted as Only Visit 1: Malaria positive. Log-binomial regression with a log link function was used to calculate the aRR and corresponding 95% CI. RR was adjusted for treatment arm (ISTp versus IPTp), maternal age, gravidity, socioeconomic status, education status, BMI, and hemoglobin at Visit 1. aRR, adjusted relative risk; BMI, body mass index; CI, confidence interval; IPTp, intermittent preventive treatment in pregnancy; ISTp, intermittent screening and treatment in pregnancy; PTB, preterm birth.

Women with at least one malaria infection during pregnancy were more likely to deliver a LBW infant (12% versus 8%, *p* = 0.024) and trended toward delivering a PTB infant (21% versus 17%, *p* = 0.054; Table 1) but were not more likely to deliver an SGA infant than malaria-negative women (17% versus 14%, *p* = 0.152; Table 1). Compared with women who were malaria negative, women who were malaria positive at Visit 1 had a higher frequency of PTB (24% versus 18%, *p* = 0.005) corresponding to an adjusted RR (aRR) of 1.30 (95% CI, 1.04–1.63, *p* = 0.021; Fig 2). Because some of these women also tested positive for malaria at later visits (scheduled or unscheduled), we repeated the analysis on women who only tested positive for malaria at Visit 1. In this restricted analysis, compared with women who were malaria negative throughout pregnancy, women with malaria only at the first antenatal visit had an aRR of 1.67 (95% CI, 1.20–2.30, *p* = 0.002) for PTB (Fig 2).

To confirm the effect of malaria status on gestational age at delivery, we analyzed gestational age at delivery by quantile regression (S3 Table). This analysis confirmed that malaria infection early in pregnancy is associated with a reduced gestational age, especially in the lower quantiles. As would be expected, it also showed a significant association between placental histopathology and gestational age at delivery in the lowest quantile (S3 Table).

There were no significant increases in risk of SGA or nonviable births in women with malaria when analyzed based on specific visits, cumulative malaria status, or placental malaria (*p* > 0.05).

### Malaria infection was associated with altered levels of inflammatory, metabolic, and angiogenic factors at each visit

At Visit 1, compared with uninfected women, malaria-infected women had significantly higher levels of CHI3L1, CRP, sICAM-1, IL-18BP, sTNFRII, and sEng and lower levels of Leptin (*p* ≤ 0.005; Table 3). At Visit 2, malaria-positive women had significantly higher levels of CRP, IL-18BP, sTNFRII, and sEng (*p* ≤ 0.005) and lower levels of PlGF and Leptin (*p* ≤ 0.005); and, at Visit 3, they had significantly higher levels of CRP, IL-18BP, sTNFRII, and sEng (*p* ≤ 0.005) and lower levels of PlGF (*p* ≤ 0.005; Table 3).

**Table 3:** The presence of malaria at each visit is associated with changes in angiogenic, metabolic, and inflammatory mediators.

Analyte by visit	Median (IQR)	Median (IQR)	
**Visit 1 (13 to 23 wks)**	**Malaria negative (*n* = 813)**	**Malaria positive (*n* = 643)**	***p* value**^#^
CHI3L1 (ng/mL)	16.55 (9.44–32.43)	19.68 (11.17–38.71)	**0.001**
CRP (ug/mL)*	2.45 (1.17–5.23)	5.69 (2.69–13.52)	**<0.001**
sICAM-1 (ng/mL)	197.64 (78.91–447.62)	235.24 (111.02–519.35)	**0.003**
IL-18 BP (ng/mL)	13.41 (9.53–18.26)	17.67 (12.36–25.76)	**<0.001**
sTNFRII (ng/mL)	2.01 (1.4–2.91)	3.78 (2.22–6.28)	**<0.001**
PlGF (pg/mL)	50.05 (29.64–83.9)	45.22 (23.43–81.69)	**0.010**
sEng (ng/mL)	2.33 (1.73–2.96)	2.78 (2.02–4.14)	**<0.001**
sFlt-1 (ng/mL)	2.00 (1.31–3.02)	2.15 (1.35–3.16)	0.123
Angptl3 (ng/mL)	15.45 (9.14–24.65)	16.93 (10.4–26.71)	0.017
Leptin (ng/mL)	7.56 (4.42–12.67)	6.53 (3.72–10.89)	**0.001**
**Visit 2 (28 to 33 wks)**	**Malaria negative (*n* = 863)**	**Malaria positive (*n* = 188)**	***p* value**
CHI3L1 (ng/mL)	17.33 (10.85–32.12)	17.67 (9.6–35.13)	0.837
CRP (ug/mL)*	2.22 (0.9–4.58)	4.52 (1.6–11.3)	**<0.001**
sICAM-1 (ng/mL)	222.16 (93.4–464.42)	212.94 (112.54–515.98)	0.417
IL-18 BP (ng/mL)	15.23 (11.93–19.6)	20.97 (15.58–27.76)	**<0.001**
sTNFRII (ng/mL)	2.40 (1.85–3.16)	2.88 (1.85–5.24)	**<0.001**
PlGF (pg/mL)	151.67 (80.02–266.04)	131.3 (55.89–247.82)	0.021
sEng (ng/mL)	2.72 (2.08–3.73)	2.93 (2.2–4.31)	**0.005**
sFlt-1 (ng/mL)	2.90 (2.01–4.07)	2.94 (2.23–4.07)	0.471
Angptl3 (ng/mL)	20.26 (11.86–31.06)	20.81 (11.89–30.54)	0.868
Leptin (ng/mL)	6.72 (3.68–11.24)	5.27 (3.38–9.32)	**0.004**
**Visit 3 (34 to 36 wks)**	**Malaria negative (*n* = 627)**	**Malaria positive (*n* = 157)**	***p* value**
CHI3L1 (ng/mL)	19.49 (11.45–34.88)	21.28 (12.19–40.21)	0.161
CRP (ug/mL)*	2.48 (1.17–4.73)	4.86 (2.07–14.85)	**<0.001**
sICAM-1 (ng/mL)	244.96 (111.09–521.38)	278.93 (152.87–480.8)	0.164
IL-18 BP (ng/mL)	15.41 (11.71–19.88)	18.91 (14.27–26.07)	**<0.001**
sTNFRII (ng/mL)	2.45 (1.88–3.09)	3.18 (2.22–5.46)	**<0.001**
PlGF (pg/mL)	82.08 (37.21–171.34)	56.55 (21.31–123.54)	**<0.001**
sEng (ng/mL)	3.93 (2.77–5.96)	4.63 (3.07–7.1)	**0.004**
sFlt-1 (ng/mL)	4.05 (2.79–5.46)	3.92 (3.03–5.16)	0.908
Angptl3 (ng/mL)	24.01 (15.32–34.58)	23.63 (14.28–32.81)	0.494
Leptin (ng/mL)	7.00 (3.87–11.87)	6.56 (3.86–9.81)	0.067

^#^Mann-Whitney analysis, adjusted *p*-value for multiple comparisons, significant if *p* ≤ 0.005 (bolded p-values).

*CRP sample numbers: Visit 1 (782;606), Visit 2 (834; 171), Visit 3 (603;147).

**Abbreviations:** Angptl3, Angiopoietin-like 3; CHI3L1, Chitinase 3-like protein-1; CRP, C-Reactive Protein; IL-18 BP, Interleukin 18 Binding Protein; IQR, interquartile range; P1GF, placental growth factor; sFlt-1, soluble Fms-like Tyrosine Kinase-1; sICAM-1, soluble Intercellular Adhesion Molecule-1; sEng, soluble Endoglin; sTNFRII, soluble Tumor Necrosis Factor receptor II

### Malaria infection before 24 weeks gestation is associated with altered longitudinal kinetics of angiogenic, metabolic, and inflammatory mediators over the course of pregnancy

**Fig 3:**
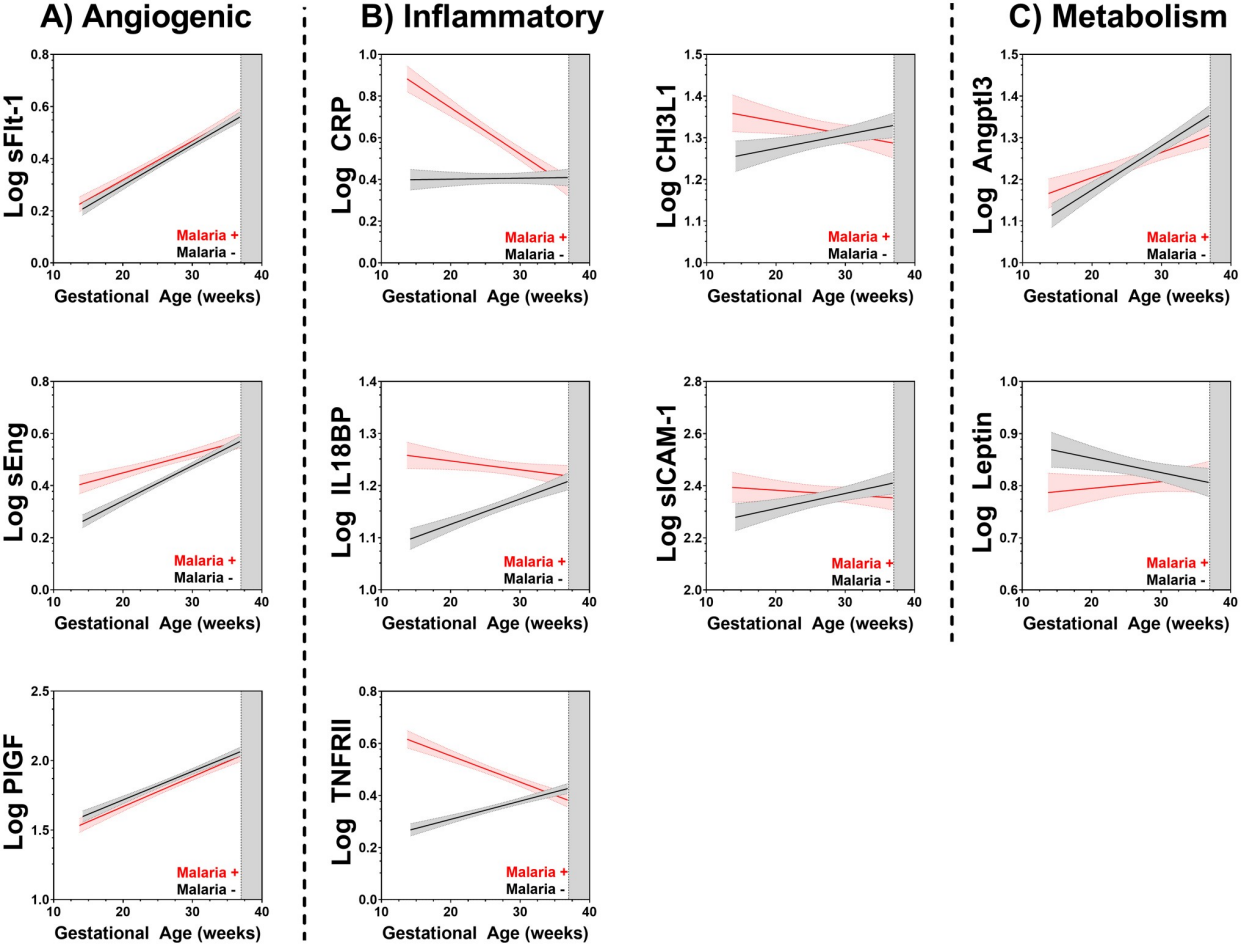
Malaria before 24 weeks gestation alters the longitudinal kinetics of angiogenic, inflammatory, and metabolic mediators over the course of pregnancy. Linear regression lines of best fit with 95% CI are represented on the graph. Malaria positive at Visit 1 (red); malaria negative at Visit 1 (black; 13 to 23 weeks gestation). Angptl3, Angiopoietin-like 3; CHI3L1, Chitinase 3-like protein-1; CI, confidence interval; CRP, C-Reactive Protein; IL18BP, Interleukin 18 Binding Protein; PlGF, placental growth factor; sEng, soluble Engdolin; sFlt-1, soluble Fms-like Tyrosine Kinase-1; sICAM-1, soluble Intercellular Adhesion Molecule-1; TNFRII, Tumor Necrosis Factor receptor II.

We used linear mixed-effects modeling to assess the kinetics of mediators across pregnancy in women based on malaria status at Visit 1 (Table 4; S4–S6 Tables), and these kinetics were visually depicted (Fig 3). This analysis demonstrated that women who were positive for malaria at Visit 1 had significantly altered kinetics of the inflammatory markers, including CRP (χ^2^ = 99.0, *p* < 0.001), CHI3L1 (χ^2^ = 21.9, *p* < 0.001), IL18-BP (χ^2^ = 44.7, *p* < 0.001), sICAM-1 (χ^2^ = 40.8, *p* < 0.001), and sTNFRII (χ^2^ = 209.2, *p* < 0.001; Fig 3, Table 4; S4 and S6 Tables) over the course of pregnancy compared with women who were malaria negative at Visit 1. Altered kinetics were also observed in women with malaria at Visit 1 for the following angiogenic and metabolic factors: sEng (χ^2^ = 38.3, *p* < 0.001), Angptl3 (χ^2^ = 13.3, *p* = 0.001), and Leptin (χ^2^ = 13.2, *p* = 0.001; Fig 3, Table 4; S5 and S6 Tables). The longitudinal kinetics of both sFlt-1 and PlGF were unaffected by malaria status at Visit 1 (*p* > 0.05; Fig 3, Table 4, S5 and S6 Tables). When analyzed based on gravidity (S7–S12 Tables), similar associations were observed between women who were primigravid, except Leptin was no longer significant (*p* = 0.075), and for women who were multigravida, except Leptin (*p* = 0.104) and sEng (*p* = 0.132) were no longer significant.

**Table 4:** Random-slope, random intercept linear mixed-effects modeling showed that malaria infection at Visit 1 (13–23 weeks) was associated with changes in inflammatory and angiogenic mediators across pregnancy. Fixed effects included a spline of gestational age, treatment group, maternal age, gravidity, socioeconomic status, educational status, BMI, and hemoglobin at Visit 1. Malaria status at Visit 1 (13–23 weeks) was added to the model, and an interaction term for gestational age and malaria status was added to a third model; then the models were compared (χ^2^, *p* value). A restricted cubic spline of gestational age was used as both main effect and in interaction terms. Extended tables including estimates for all fixed effects and parameters of model fit (AIC, BIC) are available in the supporting information (S4–S6 Tables).

Mediator	Number of subjects	Number of observations	Malaria positive at Visit 1		LR test	
			Estimate	Standard error	χ^2^	*p* Value
**sICAM-1**	1,460	3,142	0.344	0.088	40.8	**<0.001**
**CRP**	1,394	3,007	1.010	0.123	99.0	**<0.001**
**CHI3L1**	1,460	3,142	0.300	0.081	21.9	**<0.001**
**sTNFRII**	1,460	3,142	0.954	0.069	209.2	**<0.001**
**IL-18BP**	1,460	3,140	0.383	0.053	44.7	**<0.001**
**Angptl3**	1,460	3,142	0.200	0.075	13.29	**0.001**
**Leptin**	1,460	3,135	-0.062	0.064	13.16	**0.001**
**PlGF**	1,460	3,135	-0.116	0.070	2.89	0.236
**sFlt-1**	1,460	3,135	0.042	0.050	1.09	0.580
**sEng**	1,460	3,135	0.329	0.049	38.26	**<0.001**

Significant *p*-values (*p* < 0.05) are bolded.

**Abbreviations:** AIC, Akaike information criterion; Angptl3, Angiopoietin-like 3; BIC, Bayesian information criterion; BMI, body mass index; CHI3L1, Chitinase 3-like protein-1; CRP, C-Reactive Protein; IL-18BP, Interleukin 18 Binding Protein; LR, likelihood ratio; PlGF, placental growth factor; sEng, soluble Endoglin; sFlt-1, soluble Fms-like Tyrosine Kinase-1; sICAM-1, soluble Intercellular Adhesion Molecule-1; sTNFRII, soluble Tumor Necrosis Factor receptor II

## Discussion

This study assessed the longitudinal kinetics of key mediators of angiogenesis, metabolism, and inflammation over the course of pregnancy in a large cohort of women at risk of *P*. *falciparum* MIP. In these women, malaria infections detected before 24 weeks gestation altered these tightly regulated pathways that are required for placental function and healthy birth outcomes. Moreover, we show that malaria infections in early to midpregnancy (<24 weeks of gestation and before women were receiving malaria chemoprevention) were associated with PTB, a leading cause of childhood mortality [35], even if there was never another documented malaria infection during pregnancy. These observations provide insights into the pathobiology of MIP and suggest early interventions to reduce the burden of MIP may be required to prevent malaria-associated adverse birth outcomes in sub-Saharan Africa.

The study design and high prevalence of malaria in this cohort of pregnant women enabled a longitudinal assessment of the impact of MIP on key mediators over pregnancy. Inflammatory responses have previously been investigated in nonpregnant adults and children with malaria [36–41]; however, there are limited data on these mediators in MIP, especially early in pregnancy. Here, we provide first evidence that concentrations of sICAM-1, CHI3L1, and IL-18BP are increased in women with MIP before 24 weeks of gestation. These proteins play a key role in the interaction between inflammation and vascular function, and alterations in their levels are associated with an increased risk of poor birth outcomes, including PTB [18,42]. We also show that the inflammatory markers, CRP and sTNFRII, were elevated in MIP, in agreement with previous data [21,43–47]. Tight control of inflammation is required for healthy pregnancies, especially for proper placentation and fetal development, whereas dysregulated inflammation is associated with poor placental function and adverse pregnancy outcomes, including preeclampsia [48–50].

Placental vascular development requires tightly regulated expression of both angiogenic and anti-angiogenic mediators [51]. Alterations to these highly coordinated pathways can have a profound impact on placental function, fetal development, and birth outcome [22,51,52]. Here, we show that concentrations of a key anti-angiogenic factor, sEng, are significantly higher in women with MIP, in agreement with previous reports [16,25]. Moreover, we demonstrate that sEng concentrations remain significantly higher across pregnancy in women who were malaria positive at their initial visit. Increased concentrations of circulating sEng in pregnancy have previously been associated with PTB, FGR, and stillbirth [18,22,42,53,54]. sFlt-1 is another anti-angiogenic mediator; however, its role in MIP remains unclear. When measured at antenatal visits across pregnancy in women with MIP, Bostrom and colleagues showed no significant differences in peripheral levels of sFlt-1 [55], and Ruizendaal and colleagues showed a trend towards lower levels of sFlt-1 in women with peripheral malaria in the second trimester but no difference in sFlt-1 levels in the third trimester or at delivery [43]. Whereas Conroy and colleagues [16] and Muehlenbachs and colleagues [56] reported that when measured at delivery, women with smear-positive malaria have elevated peripheral and placental levels of sFlt-1. Our study showed no significant differences in levels of sFlt-1 when measured at antenatal visits across pregnancy; however, we did not evaluate levels at delivery. Further studies are required to clarify the role of sFlt-1 in MIP-associated adverse birth outcomes.

Angptl3 plays a role in both angiogenesis and lipid metabolism [57]. Here, we report that concentrations of Angptl3 increased over the course of pregnancy and that MIP in the second trimester altered its longitudinal kinetics. Our findings align with a previous study in Tanzania in which higher levels of Angptl3 before 23 weeks of pregnancy were associated with increased risk of PTB [18]. Another central metabolic protein is Leptin, which plays roles in embryonic implantation, placental endocrine function, fetal development, and immune regulation [58–60]. In this study, lower levels of Leptin were observed in women with malaria over the course of pregnancy, consistent with prior findings [43,44,61,62]. Lower Leptin levels have previously been linked to adverse birth outcomes, including PTB, miscarriage, and intrauterine growth restriction [18,58,63].

Several of the effector molecules analyzed in this study have pleiotropic effects reflecting crosstalk between the angiogenic, inflammatory, and metabolic pathways [16,51,57,59,60,64–71]. This suggests that alterations in one or more of the above mediators may trigger a cascade of events contributing to placental dysfunction and potentially PTB. Unger and colleagues demonstrated that malaria-negative women with high levels of sEng, CRP, and sFlt-1 were more likely to deliver PTB, which they attributed to other inflammatory stimuli, including infections other than malaria [42]. Several lines of evidence support the hypothesis that there are common pathways of injury that contribute to altered placental function and adverse birth outcomes due to differing initiating events, some infectious and some not [18,19]. Our data suggest that malaria infection prior to 24 weeks is one such modifiable risk factor that is associated with the alterations of these critical pathways of placental development and function. It is unknown what the key factors are, and additional mechanistic investigations will be required to define the mediators playing causal roles in the pathogenesis of adverse birth outcomes and which are a consequence of these events. Ultimately, a detailed understanding of the critical drivers of pathobiology may inform pathway-directed interventions to prevent or reduce adverse birth outcomes associated with MIP [16,24].

In this pregnancy cohort, the prevalence of PTB, as determined by ultrasound dating, was high at 20%, consistent with previous population estimates for Malawi (16%–18%) [72,73]. Our findings of an increased risk of PTB associated with malaria infection detected at 13 to 23 weeks appear to be in contrast to previous reports, suggesting that malaria infection late in pregnancy was associated with an increased risk of PTB [10,11]. Although other studies have shown that malaria infection in the first and/or second trimester is associated with LBW infants [12,74,75], these studies were largely based on the use of less rigorous methods to determine gestational age (e.g., Dubowitz, LMP versus ultrasound) and infection status (smears versus PCR diagnosis). These methods may have limited detection of submicroscopic infections earlier in pregnancy and enrolment, as well as limitations in discriminating FGR from PTB as a cause of LBW. This contention is supported by our observation that malaria infection during the second trimester was significantly associated with PTB but not SGA infants. The use of ultrasound in this study improved the accuracy of gestational age dating and, therefore, helped differentiate between causes of LBW.

This study had a number of strengths, including a prospective design that evaluated over 3,300 samples collected across pregnancy from 1,628 women at high risk of malaria early in pregnancy. This sample size provided a robust assessment of the concentrations and kinetics of critical angiogenic, metabolic, and inflammatory factors over the course of pregnancy and their relationship to MIP. Many previous efforts were conducted as cross-sectional studies with smaller sample sizes; the longitudinal nature of our study is important given the dynamic nature and tight regulation of these mediators across pregnancy. Although ultrasound dating in the first trimester is more accurate, it is difficult to achieve in settings in which women present for their first antenatal care often in their second trimester and beyond [27,76]. In Malawi, over 70% of women present after 16 weeks gestation [77]. As such, the use of second trimester ultrasound dating is a viable alternative when first trimester ultrasounds are unavailable and is reasonably accurate [27,76,78]. In our study, ultrasound dating enabled investigation into the association between MIP and PTB, a relationship that has been more challenging to define because of difficulties in obtaining reliable estimates of gestational age in low-resource settings. Despite the size of the cohort, the study was underpowered to assess the impact of malaria on risk of nonviable births. Further, the majority of malaria cases occurred in midpregnancy, so robust estimates of the impact of malaria infection occurring later in pregnancy was limited. Women who had previously taken IPTp were excluded from the study, which may have overestimated the prevalence of malaria in this cohort. All women in this study received an intervention, and, therefore, the effects observed may be underestimated for women not receiving any antimalarials. Additionally, information on other potential infections, other than HIV, was unavailable in this cohort and may also contribute to changes in these mediators of interest.

Our findings have implications for current malaria prevention strategies in pregnant women in high-transmission areas of sub-Saharan Africa. IPTp-SP from 16 weeks of gestation and insecticide-treated bednets initiated at the first antenatal visit are the current standard of care. In this study, malaria infection detected between 13 to 23 weeks gestation was already associated with alterations in angiogenic, metabolic, and inflammatory pathways, and these changes persisted throughout pregnancy. The risk of PTB remained elevated despite clearance of infection as determined by PCR. These data indicate that malaria infection frequently occurs before women present for antenatal care. Moreover, these early infections may play an important role in the development of inflammatory responses and placental dysfunction, conditions that increase the risk of PTB. WHO recommends initiating IPTp-SP as early as possible during the second trimester [79]; however, considering that almost half of the women in this study presented with malaria at enrolment, this strategy is inadequate to protect women from early malaria infections and an increased risk of PTB. Our findings are supported by Kakura and colleagues who also showed that over 50% of women had detectable parasites at their first antenatal visit [80]. Furthermore, several trials investigating strategies to reduce MIP have not reported a decrease in PTB despite a decreased prevalence of malaria over pregnancy [81–83]. Collectively, these data suggest that intervention strategies will need to be initiated earlier in pregnancy (i.e., first trimester) in order to protect women from malaria-associated adverse birth outcomes and to prevent a major cause of infant death.

Although the data from this study suggest that earlier interventions in pregnancy may be required to prevent adverse birth outcomes, such as PTB, there are currently limited interventions that can be started in the first trimester. SP is currently not recommended for administration in the first trimester [79]; folate antagonist administration is associated with neural tube defects because the neural tube closes during the first trimester [84]. Additional clinical trials are required to investigate alternative pharmacological therapeutic strategies that can be administered in the first trimester in highly endemic areas. Furthermore, additional investigations are required to understand the mechanisms underlying malaria-induced placental dysfunction and adverse birth outcomes, including PTB.

In conclusion, we found that in this cohort that MIP in midpregnancy (13–23 weeks) was associated with alterations in inflammatory, angiogenic, and metabolic pathways. These infections were associated with an increased risk of PTB. Collectively, these data support the hypothesis that MIP early in pregnancy initiates inflammatory responses and altered placental vascular function that persist throughout pregnancy and contribute to PTB. These findings suggest that intervention strategies to prevent MIP will be required earlier in pregnancy to prevent placental dysfunction and malaria-associated adverse birth outcomes.

## Supporting information

S1 Anonymized data set (XLSX)

S1 STROBE checklist STROBE, Strengthening the Reporting of Observational Studies in Epidemiology. (PDF)

S1 Table Characteristics of the assays used for marker analysis. (PDF)

S2 Table Available data (*n*) for variables and percentage of missing data. (PDF)

S3 Table Impact of malaria status on gestational age at delivery by quantile regression. (PDF)

S4 Table Multivariate linear mixed-effects modeling of the inflammatory mediators based on malaria status at Visit 1, expanded. (PDF)

S5 Table Multivariate linear mixed-effects modeling of the metabolic and angiogenic mediators based on malaria status at Visit 1, expanded. (PDF)

S6 Table Multivariate linear mixed-effects modeling comparing the null model to the addition of malaria status at Visit 1 with an interaction term assessing gestational age. (PDF)

S7 Table Multivariate linear mixed-effects modeling of the inflammatory mediators based on malaria status at Visit 1, primigravids only. (PDF)

S8 Table Multivariate linear mixed-effects modeling of the metabolic and angiogenic mediators based on malaria status at Visit 1, primigravids only. (PDF)

S9 Table Multivariate linear mixed-effects modeling comparing the null model to the addition of malaria status at Visit 1 with an interaction term assessing gestational age, primigravids only. (PDF)

S10 Table Multivariate linear mixed-effects modeling of the inflammatory mediators based on malaria status at Visit 1, multigravids only. (PDF)

S11 Table Multivariate linear mixed-effects modeling of the metabolic and angiogenic mediators based on malaria status at Visit 1, multigravids only. (PDF)

S12 Table Multivariate linear mixed-effects modeling comparing the null model to the addition of malaria status at Visit 1 with an interaction term assessing gestational age, multigravids only. (PDF)

## Abbreviations

AIC: Akaike information criterion; Angptl3, Angiopoietin-like 3
aRR: adjusted relative risk
BIC: Bayesian information criterion
BMI: body mass index
CHI3L1: Chitinase 3-like protein-1
CI: confidence interval
CRP: C-Reactive Protein
DP: dihydroartemisinin-piperaquine
FGR: fetal growth restriction
IL-18BP: Interleukin 18 Binding Protein
IPTp: intermittent preventive treatment in pregnancy
IQR: interquartile range
ISTp: intermittent screening and treatment in pregnancy
LBW: low birth weight
MIP: malaria in pregnancy
PfEMP1: *Plasmodium falciparum* erythrocyte membrane protein 1
PlGF: placental growth factor
PTB: preterm birth
RR: relative risk
sEng: soluble Endoglin
sFlt-1: soluble Fms-like Tyrosine Kinase-1
SGA: small for gestational age
sICAM-1: soluble Intercellular Adhesion Molecule-1
SP: sulfadoxine-pyrimethamine
sTNFRII: soluble Tumor Necrosis Factor receptor II
STROBE: Strengthening the Reporting of Observational Studies in Epidemiology
