# Supplementary material for: Early malaria infection, dysregulation of angiogenesis, metabolism and inflammation across pregnancy, and risk of preterm birth in Malawi: A cohort study
Source: PLoS Med. 2019 Oct 1;16(10):e1002914. doi: 10.1371/journal.pmed.1002914 (PMC6772002; doi:10.1371/journal.pmed.1002914)
Supplement: S1 Table — (PDF) [file pmed.1002914.s003.pdf]

S1 Table. Characteristics of the assays used for marker analysis. CRP and IL-18BP were analyzed by ELISA, and the other mediators were analyzed by Luminex. A control plasma sample was plated in duplicate on each plate run and the % Coefficient of Variation (CVs) are reported.

Marker	Lower Limit	Upper Limit	Mean % CV of Control Plasma
sTNFRII	31.7 pg/mL	46.3 ng/mL	7
Angptl3	578 pg/mL	843 ng/mL	6
sEndoglin	121 pg/mL	177 ng/mL	4
sFlt-1	24.0 pg/mL	35.1 ng/mL	7
Leptin	168 pg/mL	245 ng/mL	3
PlGF	4.0 pg/mL	5780 pg/mL	8
CHI3L1	4.60 ng/mL	2237 ng/mL	17
sICAM-1	30.7 ng/mL	44.7 mg/mL	10
CRP	391 ng/mL	105 mg/mL	26
IL-18BP	1.88 ng/mL	252 ng/mL	17
