# Supplementary material for: Early malaria infection, dysregulation of angiogenesis, metabolism and inflammation across pregnancy, and risk of preterm birth in Malawi: A cohort study
Source: PLoS Med. 2019 Oct 1;16(10):e1002914. doi: 10.1371/journal.pmed.1002914 (PMC6772002; doi:10.1371/journal.pmed.1002914)
Supplement: S2 Table — (PDF) [file pmed.1002914.s004.pdf]

S2 Table. Data available for each of the following variables and the percentage of missing data.

Variable	Available data, n	Percentage missing, %
For each visit (with associated plasma sample):		
Malaria Status		
Visit 1	1456	1.6
Visit 2	1051	2.7
Visit 3	784	4.2
For each woman:		
Malaria positive, peripheral blood at delivery	1391	14.6
Malaria positive, placental blood	1315	19.2
Placental histology	1374	15.6
Birth weight	1451	10.9
Gestational age at delivery	1536	5.7
Type of Delivery (spontaneous vs C-section)	1535	5.7
Delivery outcome (viable vs non-viable)	1544	5.2
