# Supplementary material for: Early malaria infection, dysregulation of angiogenesis, metabolism and inflammation across pregnancy, and risk of preterm birth in Malawi: A cohort study
Source: PLoS Med. 2019 Oct 1;16(10):e1002914. doi: 10.1371/journal.pmed.1002914 (PMC6772002; doi:10.1371/journal.pmed.1002914)
Supplement: S3 Table — (PDF) [file pmed.1002914.s005.pdf]

S3 Table. Impact of malaria status on gestational age at delivery by quantile regression[a]

	PCR Positive – Visit 1		PCR Positive – Only visit 1		PCR Positive – Visit 2		PCR Positive – Visit 3		Placental Histology		Any Malaria (PCR & Histology)	
	Estimate (SE)	p-value	Estimate (SE)	p-value	Estimate (SE)	p-value	Estimate (SE)	p-value	Estimate (SE)	p-value	Estimate (SE)	p-value
**Q1[b] (15%)**	-0.418 (0.184)	**0.02**	-0.977 (0.246)	**<0.0001**	-0.119 (0.165)	0.47	-0.247 (0.144)	0.09	-0.582 (0.229)	**0.01**	-0.498 (0.209)	**0.02**
**Q2 (50%)**	-0.292 (0.126)	**0.02**	-0.383 (0.195)	0.05	-0.027 (0.221)	0.90	-0.023 (0.173)	0.89	-0.286 (0.148)	0.05	-0.212 (0.130)	0.10
**Q3 (85%)**	-0.171 (0.136)	0.21	-0.042 (0.201)	0.83	0.089 (0.275)	0.75	-0.148 (0.186)	0.43	-0.061 (0.170)	0.72	0.001 (0.148)	0.99

[a]Quantile regression models were adjusted for treatment arm, maternal age, gravidity, socioeconomic status, education status, BMI and hemoglobin at visit 1.
[b]Quantile of gestational age at delivery; Q1 - 36.3 weeks gestation, Q2 - 38.4 weeks gestation, Q3 - 40 weeks gestation.
