# Supplementary material for: Early malaria infection, dysregulation of angiogenesis, metabolism and inflammation across pregnancy, and risk of preterm birth in Malawi: A cohort study
Source: PLoS Med. 2019 Oct 1;16(10):e1002914. doi: 10.1371/journal.pmed.1002914 (PMC6772002; doi:10.1371/journal.pmed.1002914)
Supplement: S4 Table — (PDF) [file pmed.1002914.s006.pdf]

S4 Table. Multivariate Linear Mixed Effects Modeling of the inflammatory mediators based on malaria status at visit 1, expanded.

	Inflammatory Mediators									
	sICAM-1		CRP		CHI3L1		sTNFRII		IL-18BP	
	Estimate	Std. Error	Estimate	Std. Error	Estimate	Std. Error	Estimate	Std. Error	Estimate	Std. Error
(Intercept)	5.835	0.383	1.268	0.336	3.309	0.264	1.634	0.157	2.731	0.137
**Malaria positive at visit 1[b]**	0.344	0.088	1.095	0.123	0.300	0.081	0.954	0.069	0.383	0.053
**Gestational age[c,d]**	0.015	0.006	-0.018	0.012	0.0034	0.007	0.033	0.006	0.019	0.005
**Gestational age'**	-0.007	0.007	0.023	0.013	0.004	0.008	-0.017	0.007	-0.010	0.005
**Treatment group**	0.151	0.085	0.092	0.120	0.173	0.079	0.144	0.068	0.048	0.052
**BMI at visit 1**	-0.002	0.011	0.037	0.009	0.0002	0.008	-0.005	0.004	0.0002	0.0048
**Age**	0.013	0.011	0.008	0.010	0.015	0.008	-0.008	0.004	-0.004	0.004
**Gravidity**	-0.070	0.047	-0.052	0.039	-0.054	0.032	-0.014	0.019	0.018	0.016
**Socioeconomic status**	0.002	0.014	-0.012	0.012	-0.015	0.010	-0.012	0.006	-0.014	0.005
**Education status**	-0.011	0.010	0.002	0.009	-0.006	0.007	-0.007	0.004	0.004	0.004
**Hemoglobin at visit 1**	-0.059	0.023	-0.103	0.020	-0.057	0.016	-0.070	0.009	-0.023	0.008
**Malaria visit 1*gestational age**	-0.037	0.008	-0.060	0.015	-0.032	0.008	-0.074	0.008	-0.020	0.006
**Malaria visit 1*gestational age'**	0.022	0.008	0.010	0.016	0.024	0.009	0.040	0.009	0.006	0.006
**Gestational age*treatment group**	-0.007	0.008	-0.008	0.014	-0.008	0.008	-0.009	0.008	-0.0006	0.006
**Gestational age*treatment group'**	0.009	0.008	0.008	0.016	0.001	0.009	0.005	0.008	0.0008	0.006
**Number of Subjects**	1460		1394		1460		1460		1460	
**Observations**	3142		3007		3142		3142		3140	
**LR Test**	**$\chi2$ = 40.8, p<0.001**		**$\chi2$ =99.0, p<0.001**		**$\chi2$ =21.9, p<0.001**		**$\chi2$ =209.2, p<0.001**		**$\chi2$ =44.7, P<0.001**	

[b]Malaria positive by PCR.
[c]Gestational age shifted to provide meaningful intercept.
[d]Used a restricted cubic spline of gestational age as both main effect and in interaction terms.
