# Supplementary material for: Early malaria infection, dysregulation of angiogenesis, metabolism and inflammation across pregnancy, and risk of preterm birth in Malawi: A cohort study
Source: PLoS Med. 2019 Oct 1;16(10):e1002914. doi: 10.1371/journal.pmed.1002914 (PMC6772002; doi:10.1371/journal.pmed.1002914)
Supplement: S5 Table — (PDF) [file pmed.1002914.s007.pdf]

S5 Table. Multivariate Linear Mixed Effects Modeling of the metabolic and angiogenic mediators based on malaria status at visit 1, expanded.

	**Metabolic**				**Angiogenic Mediators**					
	**Angptl3**		**Leptin**		**PlGF**		**sFlt-1**		**sEndoglin**	
	**Estimate**	**Std. Error**	**Estimate**	**Std. Error**	**Estimate**	**Std. Error**	**Estimate**	**Std. Error**	**Estimate**	**Std. Error**
(Intercept)	2.545	0.191	-1.025	0.204	2.012	0.242	1.019	0.172	1.447	0.191
**Malaria positive at visit 1[a]**	0.200	0.075	-0.062	0.064	-0.116	0.070	0.042	0.050	0.329	0.049
**Gestational age[b,c]**	0.021	0.007	-0.021	0.006	0.195	0.007	0.019	0.004	-0.015	0.004
**Gestational age'**	0.005	0.008	0.016	0.006	-0.177	0.007	0.025	0.004	0.057	0.004
**Treatment group**	0.048	0.073	-0.076	0.062	-0.006	0.068	0.042	0.048	0.009	0.048
**BMI at visit 1**	0.003	0.005	0.108	0.006	-0.018	0.007	-0.010	0.005	0.003	0.005
**Age**	0.008	0.006	-0.006	0.006	-0.005	0.007	-0.009	0.005	-0.003	0.006
**Gravidity**	-0.054	0.023	-0.020	0.025	0.024	0.029	-0.021	0.021	-0.068	0.023
**Socioeconomic status**	0.018	0.007	0.039	0.008	0.011	0.009	0.003	0.006	0.001	0.007
**Education status**	0.008	0.005	0.002	0.005	0.016	0.006	0.007	0.004	0.0004	0.005
**Hemoglobin at visit 1**	-0.015	0.011	0.071	0.012	0.097	0.015	-0.004	0.010	-0.036	0.011
**Malaria visit 1*gestational age**	-0.022	0.009	0.005	0.007	0.014	0.009	-0.004	0.004	-0.023	0.005
**Malaria visit 1*gestational age'**	0.013	0.010	0.006	0.008	-0.014	0.009	0.004	0.004	0.011	0.005
**Gestational age*treatment group**	-0.002	0.009	0.001	0.007	-0.003	0.008	0.001	0.004	-0.0005	0.005
**Gestational age*treatment group'**	-0.0003	0.010	-0.002	0.008	0.004	0.009	-0.005	0.004	-0.001	0.005
**Number of Subjects**	1460		1460		1460		1460		1460	
**Observations**	3142		3135		3135		3135		3135	
**LR Test**	**$\chi2$=13.29, p=0.001**		**$\chi2$=13.16, p=0.001**		**$\chi2$=2.89, p=0.236**		**$\chi2$= 1.09, p=0.580**		**$\chi2$= 38.26, p < 0.001**	

[a]Malaria positive by PCR.
[b]Gestational age shifted to provide meaningful intercept.
[c]Used a restricted cubic spline of gestational age as both main effect and in interaction terms.
