# Supplementary material for: Early malaria infection, dysregulation of angiogenesis, metabolism and inflammation across pregnancy, and risk of preterm birth in Malawi: A cohort study
Source: PLoS Med. 2019 Oct 1;16(10):e1002914. doi: 10.1371/journal.pmed.1002914 (PMC6772002; doi:10.1371/journal.pmed.1002914)
Supplement: S6 Table — (PDF) [file pmed.1002914.s008.pdf]

S6 Table. Multivariate Linear Mixed Effects Modeling comparing the null model to the addition of malaria status at Visit 1 with an interaction term assessing gestational age.

Mediator	Model	AIC	BIC	$\chi 2$	Chi Df	P value
**sEng**	Null model	4836.1	4932.9			
	Model with malaria term	4822.9	4925.8	15.18	1	**<0.001**
	Model with interaction term	4788.7	4903.6	38.26	2	**<0.001**
**PlGF**	Null model	7227.4	7324.2			
	Model with malaria term	7229.1	7332.0	0.29	1	0.593
	Model with interaction term	7230.2	7345.2	2.89	2	0.236
**sFlt-1**	Null model	4063.5	4160.3			
	Model with malaria term	4065.5	4168.4	0.009	1	0.925
	Model with interaction term	4068.4	4183.4	1.09	2	0.580
**Angptl3**	Null model	6519.1	6615.9			
	Model with malaria term	6520.9	6623.8	0.167	1	0.683
	Model with interaction term	6511.6	6626.6	13.29	2	**0.001**
**Leptin**	Null model	6047.2	6144.0			
	Model with malaria term	6048.9	6151.8	0.29	1	0.593
	Model with interaction term	6039.8	6154.7	13.16	2	**0.001**
**sICAM-1**	Null model	8112.6	8209.5			
	Model with malaria term	8114.6	8217.5	0.0016	1	0.968
	Model with interaction term	8077.9	8192.9	40.75	2	**<0.001**
**CRP**	Null model	9435	9531.1			
	Model with malaria term	9387.4	9489.5	49.59	1	**<0.001**
	Model with interaction term	9291.7	9399.8	99.0	1	**<0.001**
**CHI3L1**	Null model	7286.5	7383.3			
	Model with malaria term	7288.5	7391.4	0.0087	1	0.926
	Model with interaction term	7270.6	7385.6	21.93	2	**<0.001**
**sTNFRII**	Null model	5883.3	5980.2			
	Model with malaria term	5859	5961.8	26.38	1	**<0.001**
	Model with interaction term	5653.8	5768.8	209.18	2	**<0.001**
**IL-18BP**	Null model	4282.3	4379.2			
	Model with malaria term	4248	4350.9	36.32	1	**<0.001**
	Model with interaction term	4207.3	4322.3	44.70	2	**<0.001**
