# Supplementary material for: Early malaria infection, dysregulation of angiogenesis, metabolism and inflammation across pregnancy, and risk of preterm birth in Malawi: A cohort study
Source: PLoS Med. 2019 Oct 1;16(10):e1002914. doi: 10.1371/journal.pmed.1002914 (PMC6772002; doi:10.1371/journal.pmed.1002914)
Supplement: S7 Table — (PDF) [file pmed.1002914.s009.pdf]

S7 Table. Multivariate Linear Mixed Effects Modeling of the inflammatory mediators based on malaria status at Visit 1, primigravids only

	Inflammatory Mediators									
	sICAM-1		CRP		CHI3L1		sTNFRII		IL18BP	
	Estimate	Std. Error	Estimate	Std. Error	Estimate	Std. Error	Estimate	Std. Error	Estimate	Std. Error
(Intercept)	5.789	0.742	1.471	0.736	2.483	0.554	1.379	0.318	2.824	0.273
Malaria positive at visit 1[a]	0.401	0.143	1.281	0.211	0.220	0.130	1.036	0.107	0.490	0.083
Gestational age[b,c]	0.008	0.012	-0.029	0.027	-0.004	0.014	0.026	0.014	0.014	0.010
Gestational age'	-0.002	0.013	0.045	0.030	0.017	0.015	-0.011	0.015	-0.005	0.010
Treatment group	0.187	0.134	0.167	0.200	0.229	0.123	0.128	0.102	-0.025	0.080
BMI at visit 1	-0.021	0.019	0.015	0.019	0.007	0.014	-0.003	0.008	-0.007	0.007
Age	0.040	0.027	0.026	0.026	0.036	0.020	0.016	0.011	0.005	0.010
Socioeconomic status	-0.009	0.025	-0.027	0.026	-0..033	0.018	-0.028	0.010	-0.018	0.009
Education status	-0.012	0.019	-0.011	0.018	-0.003	0.014	-0.006	0.008	0.001	0.007
Hemoglobin at visit 1	-0.065	0.037	-0.112	0.036	-0.028	0.027	-0.083	0.015	-0.023	0.013
Malaria visit 1*gestational age	-0.031	0.013	-0.042	0.029	-0.024	0.015	-0.080	0.015	-0.031	0.010
Malaria visit 1*gestational age'	0.017	0.014	-0.029	0.032	0.013	0.016	0.035	0.016	0.014	0.011
Gestational age*treatment group	-0.020	0.013	-0.010	0.028	-0.008	0.014	-0.010	0.014	0.011	0.010
Gestational age*treatment group'	0.018	0.013	-0.004	0.032	-0.007	0.016	0.001	0.016	-0.011	0.011
Number of Subjects	500		435		500		500		500	
Observations	1067		936		1067		1067		1067	
LR Test	**$\chi2$=9.85, p=0.007**		**$\chi2$=39.6, p<0.001**		**$\chi2$=7.42, p=0.024**		**$\chi2$=89.30, p<0.001**		**$\chi2$=23.29, p<0.001**	

[a]Malaria positive by PCR. [b]Gestational age shifted to provide meaningful intercept. [c]Used a restricted cubic spline of gestational age as both main effect and in interaction terms.
