# Supplementary material for: Early malaria infection, dysregulation of angiogenesis, metabolism and inflammation across pregnancy, and risk of preterm birth in Malawi: A cohort study
Source: PLoS Med. 2019 Oct 1;16(10):e1002914. doi: 10.1371/journal.pmed.1002914 (PMC6772002; doi:10.1371/journal.pmed.1002914)
Supplement: S8 Table — (PDF) [file pmed.1002914.s010.pdf]

**S8 Table**. Multivariate Linear Mixed Effects Modeling of the metabolic and angiogenic mediators based on malaria status at Visit 1, primigravids only

| | **Metabolic** | | | | **Angiogenic Mediators** | | | | | |
|---|---|---|---|---|---|---|---|---|---|---|
| | **Angptl3** | | **Leptin** | | **PlGF** | | **sFlt1** | | **sEndoglin** | |
| | **Estimate** | **Std. Error** | **Estimate** | **Std. Error** | **Estimate** | **Std. Error** | **Estimate** | **Std. Error** | **Estimate** | **Std. Error** |
| **(Intercept)** | 2.923 | 0.365 | -0.500 | 0.405 | 2.802 | 0.500 | 1.322 | 0.330 | 1.776 | 0.417 |
| **Malaria positive at visit 1[a]** | 0.110 | 0.109 | -0.070 | 0.102 | -0.206 | 0.115 | -0.002 | 0.079 | 0.732 | 0.091 |
| **Gestational age[b,c]** | 0.023 | 0.014 | -0.019 | 0.012 | 0.193 | 0.014 | 0.010 | 0.007 | -0.003 | 0.009 |
| **Gestational age'** | 0.011 | 0.015 | 0.016 | 0.013 | -0.195 | 0.015 | 0.036 | 0.007 | 0.054 | 0.009 |
| **Treatment group** | 0.081 | 0.104 | -0.183 | 0.097 | -0.105 | 0.109 | 0.018 | 0.076 | 0.079 | 0.086 |
| **BMI at visit 1** | -0.001 | 0.009 | 0.086 | 0.010 | -0.026 | 0.013 | -0.012 | 0.009 | 0.006 | 0.011 |
| **Age** | 0.007 | 0.013 | 0.001 | 0.015 | -0.005 | 0.018 | -0.016 | 0.012 | -0.018 | 0.015 |
| **Socioeconomic status** | 0.014 | 0.012 | 0.050 | 0.013 | 0.002 | 0.017 | 0.018 | 0.011 | 0.010 | 0.014 |
| **Education status** | -0.002 | 0.009 | -0.016 | 0.010 | 0.014 | 0.012 | 0.006 | 0.008 | -0.013 | 0.010 |
| **Hemoglobin at visit 1** | -0.034 | 0.018 | 0.065 | 0.020 | 0.066 | 0.025 | -0.004 | 0.016 | -0.059 | 0.021 |
| **Malaria visit 1*gestational age** | -0.014 | 0.015 | -0.005 | 0.013 | 0.017 | 0.016 | -0.001 | 0.007 | -0.059 | 0.010 |
| **Malaria visit 1*gestational age'** | 0.002 | 0.016 | 0.017 | 0.014 | -0.000 | 0.017 | -0.000 | 0.007 | 0.033 | 0.010 |
| **Gestational age*treatment group** | -0.012 | 0.014 | 0.011 | 0.013 | 0.006 | 0.015 | 0.006 | 0.007 | -0.005 | 0.009 |
| **Gestational age*treatment group'** | 0.008 | 0.016 | -0.006 | 0.014 | 0.005 | 0.016 | -0.011 | 0.007 | -0.002 | 0.009 |
| **Number of Subjects** | 500 | | 500 | | 500 | | 500 | | 500 | |
| **Observations** | 1067 | | 1062 | | 1062 | | 1062 | | 1062 | |
| **LR Test** | **$\chi2$=6.37, p=0.041** | | **$\chi2$=5.17, p=0.08** | | **$\chi2$=5.41, p=0.067** | | **$\chi2$=0.05, p=0.975** | | **$\chi2$=51.0, p<0.001** | |

[a]Malaria positive by PCR. [b]Gestational age shifted to provide meaningful intercept. [c]Used a restricted cubic spline of gestational age as both main effect and in interaction terms.
