# Supplementary material for: Early malaria infection, dysregulation of angiogenesis, metabolism and inflammation across pregnancy, and risk of preterm birth in Malawi: A cohort study
Source: PLoS Med. 2019 Oct 1;16(10):e1002914. doi: 10.1371/journal.pmed.1002914 (PMC6772002; doi:10.1371/journal.pmed.1002914)
Supplement: S9 Table — (PDF) [file pmed.1002914.s011.pdf]

S9 Table. Multivariate Linear Mixed Effects Modeling comparing the null model to the addition of malaria status at Visit 1 with an interaction term assessing gestational age, primigravids only.

Mediator	Model	AIC	BIC	$\chi 2$	Chi Df	P value
**sEng**	Null model	1948.4	2022.9			
	Model with malaria term	1932.1	2011.6	18.28	1	**<0.001**
	Model with interaction term	1885.1	1974.5	51.04	2	**<0.001**
**PlGF**	Null model	2603.1	2677.6			
	Model with malaria term	2603.6	2683.1	1.50	1	0.221
	Model with interaction term	2602.2	2691.6	5.41	2	0.067
**sFlt-1**	Null model	1383.4	1458.0			
	Model with malaria term	1385.4	1464.9	0.063	1	0.801
	Model with interaction term	1389.3	1478.7	0.051	2	0.975
**Angptl3**	Null model	2143.4	2218.0			
	Model with malaria term	2144.4	2224.0	0.93	1	0.334
	Model with interaction term	2142.1	2231.6	6.37	2	**0.041**
**Leptin**	Null model	2111.2	2185.7			
	Model with malaria term	2112.6	2192.1	0.59	1	0.443
	Model with interaction term	2111.4	2200.8	5.17	2	0.075
**sICAM-1**	Null model	2749.4	2824.0			
	Model with malaria term	2749.8	2829.4	1.65	1	0.199
	Model with interaction term	2743.9	2833.4	9.85	2	**0.007**
**CRP**	Null model	3141.3	3213.9			
	Model with malaria term	3104.3	3181.8	39.01	1	**<0.001**
	Model with interaction term	3068.7	3155.9	39.57	2	**<0.001**
**CHI3L1**	Null model	2531.3	2605.9			
	Model with malaria term	2533.3	2612.9	0.03	1	0.867
	Model with interaction term	2529.9	2619.4	7.42	2	**0.024**
**sTNFRII**	Null model	2166.5	2241.1			
	Model with malaria term	2137.8	2217.3	30.75	1	**<0.001**
	Model with interaction term	2052.5	2142.0	89.30	2	**<0.001**
**IL-18BP**	Null model	1549.0	1623.5			
	Model with malaria term	1531.4	1610.9	19.57	1	**<0.001**
	Model with interaction term	1512.1	1601.6	23.29	2	**<0.001**
