# Supplementary material for: Early malaria infection, dysregulation of angiogenesis, metabolism and inflammation across pregnancy, and risk of preterm birth in Malawi: A cohort study
Source: PLoS Med. 2019 Oct 1;16(10):e1002914. doi: 10.1371/journal.pmed.1002914 (PMC6772002; doi:10.1371/journal.pmed.1002914)
Supplement: S10 Table — (PDF) [file pmed.1002914.s012.pdf]

S10 Table. Multivariate Linear Mixed Effects Modeling of the inflammatory mediators based on malaria status at Visit 1, multigravids only

	Inflammatory Mediators									
	sICAM-1		CRP		CHI3L1		sTNFRII		IL18BP	
	Estimate	Std. Error	Estimate	Std. Error	Estimate	Std. Error	Estimate	Std. Error	Estimate	Std. Error
(Intercept)	5.540	0.503	0.754	0.406	3.401	0.332	1.388	0.195	2.493	0.176
Malaria positive at visit 1[a]	0.227	0.112	0.914	0.147	0.308	0.103	0.719	0.085	0.243	0.066
Gestational age[b,c]	0.016	0.007	-0.016	0.013	0.006	0.008	0.036	0.007	0.022	0.006
Gestational age'	-0.007	0.008	-0.017	0.015	-0.001	0.009	-0.020	0.008	-0.013	0.006
Treatment group	0.117	0.106	0.037	0.140	0.146	0.098	0.147	0.081	0.095	0.063
BMI at visit 1	0.005	0.014	0.042	0.011	-0.004	0.009	-0.007	0.005	0.002	0.005
Age	<0.001	0.009	0.004	0.007	0.012	0.006	-0.007	0.003	<0.001	0.003
Socioeconomic status	0.0006	0.018	-0.009	0.014	-0.007	0.012	-0.008	0.007	-0.014	0.006
Education status	-0.008	0.012	0.010	0.009	-0.007	0.008	-0.009	0.004	0.003	0.004
Hemoglobin at visit 1	-0.041	0.031	-0.074	0.025	-0.065	0.020	-0.054	0.012	-0.014	0.011
Malaria visit 1*gestational age	-0.033	0.010	-0.064	0.018	-0.034	0.011	-0.060	0.010	-0.008	0.007
Malaria visit 1*gestational age'	0.021	0.011	0.027	0.019	0.027	0.012	0.036	0.010	-0.003	0.008
Gestational age*treatment group	<0.001	0.009	-0.005	0.017	-0.008	0.010	-0.009	0.009	-0.007	0.007
Gestational age*treatment group'	0.003	0.010	0.011	0.018	0.006	0.011	0.006	0.010	0.007	0.007
Number of Subjects	960		959		960		960		960	
Observations	2075		2071		2075		2075		2073	
LR Test	**χ2=20.04, p<0.001**		**χ2=46.16, p<0.001**		**χ2=12.52, p=0.002**		**χ2=79.80, p<0.001**		**χ2=13.92, p<0.001**	

[a]Malaria positive by PCR. [b]Gestational age shifted to provide meaningful intercept. [c]Used a restricted cubic spline of gestational age as both main effect and in interaction terms.
