# Supplementary material for: Early malaria infection, dysregulation of angiogenesis, metabolism and inflammation across pregnancy, and risk of preterm birth in Malawi: A cohort study
Source: PLoS Med. 2019 Oct 1;16(10):e1002914. doi: 10.1371/journal.pmed.1002914 (PMC6772002; doi:10.1371/journal.pmed.1002914)
Supplement: S11 Table — (PDF) [file pmed.1002914.s013.pdf]

S11 Table. Multivariate linear mixed effects modeling of the metabolic and angiogenic mediators based on malaria status at Visit 1, multigravids only

	Metabolic				Angiogenic Mediators					
	Angptl3		Leptin		PlGF		sFlt1		sEndoglin	
	Estimate	Std. Error	Estimate	Std. Error	Estimate	Std. Error	Estimate	Std. Error	Estimate	Std. Error
(Intercept)	2.077	0.249	-1.142	0.263	1.944	0.306	0.431	0.223	0.607	0.221
Malaria positive at visit 1[a]	0.245	0.098	-0.040	0.080	-0.041	0.085	0.038	0.063	-0.014	0.053
Gestational age[b,c]	0.020	0.009	-0.020	0.007	0.198	0.008	0.023	0.004	-0.019	0.004
Gestational age'	0.004	0.010	0.014	0.007	-0.172	0.008	0.021	0.004	0.057	0.004
Treatment group	0.021	0.093	-0.011	0.076	0.041	0.080	0.063	0.060	-0.035	0.050
BMI at visit 1	0.004	0.006	0.118	0.007	-0.016	0.008	-0.009	0.006	0.020	0.006
Age	0.006	0.004	-0.012	0.005	-0.007	0.005	-0.002	0.004	-0.002	0.004
Socioeconomic status	0.020	0.009	0.033	0.009	0.014	0.011	-0.008	0.008	-0.001	0.008
Education status	0.013	0.006	0.010	0.006	0.016	0.007	0.007	0.005	0.008	0.005
Hemoglobin at visit 1	0.011	0.015	0.066	0.016	0.104	0.019	0.019	0.014	0.021	0.014
Malaria visit 1*gestational age	-0.027	0.012	0.009	0.009	0.009	0.010	-0.004	0.006	0.005	0.006
Malaria visit 1*gestational age'	0.018	0.013	-0.001	0.010	-0.013	0.011	0.003	0.006	-0.010	0.006
Gestational age*treatment group	0.003	0.011	-0.005	0.008	-0.008	0.010	-0.002	0.005	0.001	0.005
Gestational age*treatment group'	-0.004	0.012	0.002	0.009	0.002	0.010	-0.002	0.006	0.000	0.005
Number of Subjects	960		960		960		960		960	
Observations	2075		2073		2073		2073		2073	
LR Test	**χ2=10.14, p=0.006**		**χ2=4.53, p=0.104**		**χ2=1.84, p=0.399**		**χ2=0.62, p=0.733**		**χ2= 4.05, p=0.132**	

[a]Malaria positive by PCR. [b]Gestational age shifted to provide meaningful intercept. [c]Used a restricted cubic spline of gestational age as both main effect and in interaction terms.
