# Supplementary material for: Early malaria infection, dysregulation of angiogenesis, metabolism and inflammation across pregnancy, and risk of preterm birth in Malawi: A cohort study
Source: PLoS Med. 2019 Oct 1;16(10):e1002914. doi: 10.1371/journal.pmed.1002914 (PMC6772002; doi:10.1371/journal.pmed.1002914)
Supplement: S12 Table — (PDF) [file pmed.1002914.s014.pdf]

S12 Table. Multivariate linear mixed effects modeling comparing the null model to the addition of malaria status at Visit 1 with an interaction term assessing gestational age, multigravids only.

Mediator	Model	AIC	BIC	$\chi2$	Chi Df	P value
**sEng**	Null model	2692.6	2777.2			
	Model with malaria term	2694.4	2784.6	0.23	1	0.628
	Model with interaction term	2694.4	2795.8	4.05	2	0.132
**PlGF**	Null model	4613.9	4698.4			
	Model with malaria term	4615.8	4706.0	0.09	1	0.768
	Model with interaction term	4618.0	4719.4	1.84	2	0.399
**sFlt-1**	Null model	2659.6	2744.1			
	Model with malaria term	2661.6	2751.8	<0.01	1	0.947
	Model with interaction term	2664.9	2766.4	0.62	2	0.733
**Angptl3**	Null model	4376.8	4461.4			
	Model with malaria term	4378.7	4468.9	0.11	1	0.741
	Model with interaction term	4372.6	4474.0	10.14	2	**0.006**
**Leptin**	Null model	3932.0	4016.6			
	Model with malaria term	3932.6	4022.8	1.47	1	0.226
	Model with interaction term	3932.0	4033.5	4.53	2	0.104
**sICAM-1**	Null model	5362.9	5447.5			
	Model with malaria term	5364.0	5454.2	0.86	1	0.354
	Model with interaction term	5348.0	5449.5	20.04	2	**<0.001**
**CRP**	Null model	6243.5	6328.1			
	Model with malaria term	6230.4	6320.5	15.16	1	**<0.001**
	Model with interaction term	6188.2	6289.6	46.16	2	**<0.001**
**CHI3L1**	Null model	4762.5	4847.1			
	Model with malaria term	4764.5	4854.7	<0.01	1	0.992
	Model with interaction term	4756.0	4857.5	12.52	2	**0.002**
**sTNFRII**	Null model	3606.3	3690.9			
	Model with malaria term	3603.0	3693.2	5.31	1	**0.021**
	Model with interaction term	3527.2	3628.7	79.80	2	**<0.001**
**IL-18BP**	Null model	2727.9	2812.4			
	Model with malaria term	2713.9	2804.1	15.95	1	**<0.001**
	Model with interaction term	2704.0	2805.5	13.92	2	**<0.001**
